# Template-directed synthesis and characterization of microstructured ceramic Ce/ZrO_2_@SiO_2_ composite tubes

**DOI:** 10.3762/bjnano.5.126

**Published:** 2014-07-25

**Authors:** Jörg J Schneider, Meike Naumann

**Affiliations:** 1 Eduard-Zintl Institut für Anorganische und Physikalische Chemie, Technische Universität Darmstadt, Alarich-Weiss-Str.12, 64287 Darmstadt, Germany

**Keywords:** electrospinning, exotemplating, nanostructured solid solution, sol–gel chemistry, Stoeber process, ternary oxide

## Abstract

An exo-templating synthesis process using polymeric fibers and inorganic sol particles deposited onto structured one-dimensional objects is presented. In particular, CeO_2_/ZrO_2_@SiO_2_ composite tubes were synthesized in a two-step procedure by using electrospun polystyrene fibers as fiber template. First, a sol–gel approach based on an exo-templating technique was employed to obtain polystyrene(PS)/SiO_2_ composite fibers. These composite fibers were subsequently covered by spray-coating with ceria and zirconia sol solutions. After drying and final calcination of the green body composites, the PS polymer template was removed, and composite tubes of the composition CeO_2_/ZrO_2_@SiO_2_ were obtained. The SiO_2_/ZrO_2_/CeO_2_ microtubes, which consist of interconnected silica particles, are held together by ceria and zirconia deposits formed during the thermal treatment process. These microtubes are mainly located in the pendentive connecting the individual spherical silica particles and glue them together. The composition and crystallinity of this material connecting the individual silica particles contains the elements Ce and Zr and O as mixed oxide solid solution identified by XRD, Raman and high-resolution TEM and EFTEM. High-resolution microscopy techniques allowed for an elemental mapping on the surface of the silica host structure and determination of the O, Zr and Ce elemental distribution with nm precision.

## Introduction

Ceria, CeO_2_, is well-known for its unique acid–base and redox properties, which has led to numerous applications in catalysis, energy related studies (e.g., for solid fuel cells), in gas sensor technologies and in biochemistry [1–3]. Its high oxygen storage/release capacity is a result of the high reducibility of Ce^4+^ to Ce^3+^, which relies on the high mobility of oxygen ions inside the ceria lattice [2–3]. Pure ceria, however, has a low thermal stability and is prone to sintering at high temperatures, which leads to its deactivation as a catalyst. The addition of a defined amount of zirconia enhances its active surface area, thermal stability, and oxygen storage capacity [3–4]. Such ceria/zirconia solid composite materials represent solid solutions in which the Ce/Zr ratio can be adjusted over a wide range. It has been shown that Ce*_x_*Zr_1−_*_x_*O_2_ solid solutions have enhanced structural and textural properties, improved thermal stability as well as redox properties [5–8]. The transformation into ordered crystalline mesoporous structures of composition Ce*_x_*Zr_1−_*_x_*O_2_ (*x* = 0.4–0.8) has also been shown [9]. In these solid solutions ultrafine zirconia allows for a better mechanical behavior, which facilitates enhanced fracture toughness with critical stress intensities as high as 20 MPa m^1/2^ [10]. With respect to catalysis, solid solutions of the composition Ce*_x_*Zr_1−_*_x_*O_2_ have been studied for the generation of hydrogen (e.g., in fuel cell applications), CO_2_ reforming (e.g., for production of synthesis gas), direct methane oxidation (e.g., for solid oxide fuel cells), SOFCs [3,5] and electrochromic smart window applications [11]. Adding silica as a support enhances the oxygen storage capacity (OSC) of such ceria–zirconia composite materials [2,4]. Besides synthetic methods such as the thermal decomposition of precursors [12], co-precipitation for the preparation of powders [13], impregnation [9], dip-coating [5], or hydrothermal synthesis [4], sol–gel synthesis routes have been widely employed for the preparation of Ce*_x_*Zr_1−_*_x_*O_2_ solid solutions [14]. Pure aqueous sols or sols stabilized by the addition of organics, e.g., surfactants, allow for the stabilization of reactive pre-ceramic compositions in solution. High-temperature annealing steps have to be employed to obtain the solid ceramic solutions from such a solution processing route of the precursors.

Herein, we report on a newly developed synthetic process based on a combination of electrospinning and exotemplating leading to hollow CeO_2_/ZrO_2_@SiO_2_ composite tubes. Firstly, after electrospinning of polystyrene fibers, the fibers were covered by an exotemplating step with a sol solution containing monodisperse silica particles obtained from a Stoeber process. This is followed by the addition of ceria and zirconia nanoparticles, both of which are obtained from stable sol solutions by spray-coating onto the former material. The overall process yields ceramic microtubes of the composition Ce_0.13_/Zr_0.87_O_2_@SiO_2_ after calcination when a 1:7 molar ratio of Ce:Zr was employed. The obtained tubes are strengthened by the defined zirconia/ceria composition compared to pure SiO_2_ tubes obtained under the identical exo-templating conditions. It is shown that a stable and intimate ceramic nanocrystalline interface exists between the Stoeber particles composing the silica tubes and the mixed ceria/zirconia solid solution of the composition Ce_0.13_/Zr_0.87_O_2_ which is connecting the SiO_2_ particles as a ceramic binder. Based on these findings future studies may investigate the effect of this phase on the hardness, viz. the mechanical properties.

## Results and Discussion

### Synthesis of hollow SiO_2_ microtubes by exo-templating

**Figure 1:**
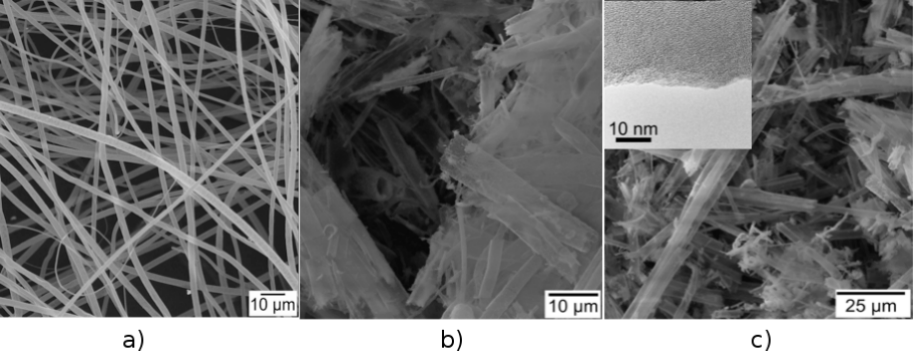
a) SEM image of electrospun PS template fibers; b) and c) SEM at two different magnifications of microsized silica tubes after calcination (750 °C, 4 h). The silica tubes are obtained by using electrospun PS fibers as exotemplates, functionalizing them by an O_2_ plasma treatment followed by coverage with a layer of Stoeber sol particles. The average diameter of the silica tubes is ca. 2 µm. However, smaller tubes exist due to the non-uniform electrospinning process yielding the PS tubes. The inset in c) shows a HRTEM picture of the silica side wall of an individual tube depicting its amorphous character.

Tubular structures of ternary oxide CeO_2_/ZrO_2_@SiO_2_ were synthesized by using a multistep synthesis route. In the first step, dense fibrous mats of polystyrene (PS) fibers were obtained by an electrospinning process [15]. An inorganic/polymer composite, PS/silica, was synthesized by using these PS template fibers as substrate by depositing a Stoeber particle sol solution on the surface of this polymeric fibrous material. This solution contains spherical silica particles in a narrow size range of 150 ± 10 nm. For an effective tethering of these uniform silica particles on the PS fiber surface, the as-obtained PS fibers were surface-functionalized in a reactive oxygen plasma atmosphere (rf plasma, 60 W, 20% O_2_) prior to the addition and anchoring of the silica particles to these surface-functionalized groups. This ensures a dense and covalent linking of the particles to the PS surface. Calcination removes the PS fiber template the silica particles are attached to and subsequently converts the dense composite fiber morphology into a hollow tubular structure, which results in the formation of pure silica tubes. Figure 1 shows SEM images of the pure polymer PS template fibers and the obtained bundles of as-prepared ceramic silica tubes after calcination and removal of the electrospun PS fiber template (750 °C, 4 h). Their tubular structure consists of densely packed agglomerated silica particles (see Figure 1). A high-resolution TEM (HRTEM) study reveals the amorphous nature of the sidewalls of the silica tubes.

### Synthesis and characterization of ternary ceria/zirconia@silica composite microtubes

**Figure 2:**
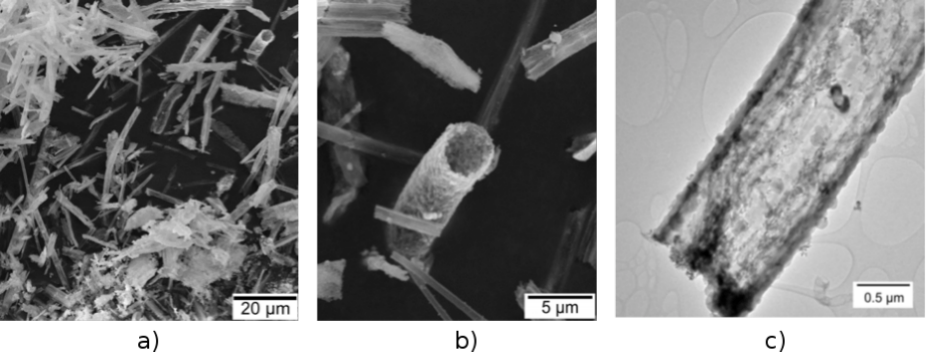
a), b): SEM images of the final ternary ceramic oxide composite tubes CeO_2_/ZrO_2_@SiO_2_, obtained at 750 °C, 4 h at two different magnifications; c) TEM image showing the hollow structure of the CeO_2_/ZrO_2_@SiO_2_ microtubes.

The PS/silica composite fiber material with its inner PS core was used as an exo-template for the deposition of an ethanolic sol solution containing ceria and zirconia sol precursor aggregates by a spray-coating procedure. After impregnation with this sol the initial PS fiber template was selectively removed from the PS/silica/ceria/zirconia composite by calcination. Figure 2 shows SEM images of the obtained ceria/zirconia@silica ceramic composite tubes after the final calcination at 750 °C. The hollow tube morphology obtained by this process is clearly visible, which indicates the successful thermal removal of the PS template. There is only a minor amount of bulk ceria and/or zirconia material aside from the microtubular composite tubes, which hints at an almost preferential deposition of the ceria and zirconia sol solutions on the plasma-functionalized silica tubes during the spray-coating process. This is a viable indication for the successful gas phase plasma functionalization of the surface of the SiO_2_, which facilitates a very effective anchoring of the sol particles. According to the microsized structure of the composite tubes of the composition Ce_0.13_/Z_0.87_O_2_@SiO_2_ (Ce/Zr ratio 1:7) the BET surface area was determined to be 30 m^2^/g^−1^. This is considerably higher than samples of ultrafine powders with a particle size of 80 nm of the composition Ce_0.12_/Zr_0.88_O_2_ [11].

**Scheme 1:**
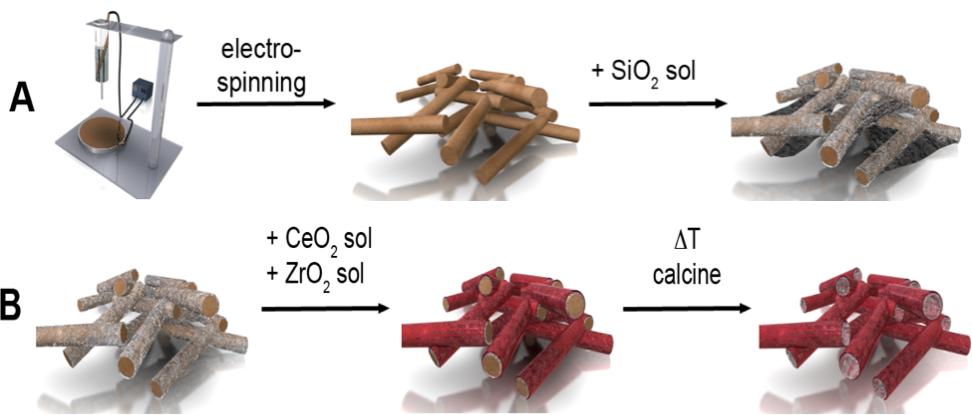
Reaction sequence starting from an electrospinning process yielding PS fibers, followed by the subsequent deposition of silica, ceria and zirconia sol formulations and the drying of the deposits. The final calcination removes the inside PS template fibers (shown in brown) to yield ceramic oxide composite tubes of the composition ceria/zirconia@silica.

Scheme 1 shows the series of synthetic steps which first lead to the PS/silica tubes exotemplate (A) and after sol–gel infiltration to the final CeO_2_/ZrO_2_@SiO_2_ composite tubes (B).

**Figure 3:**
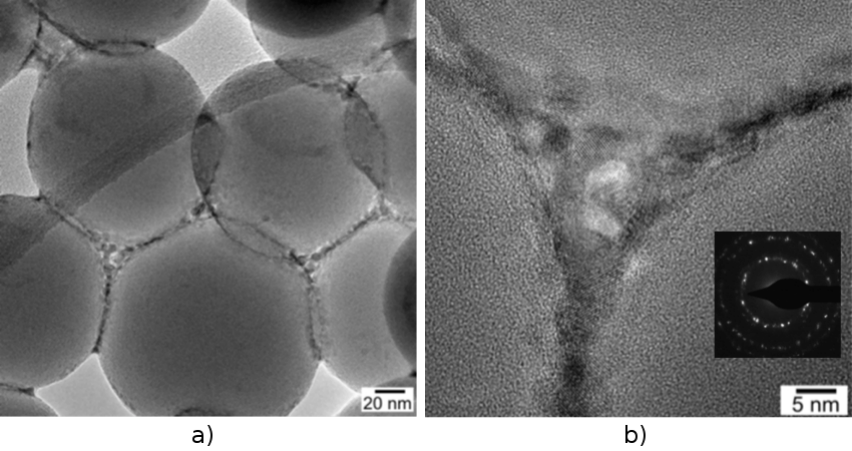
TEM images displaying the particulate character of the ternary oxide microtubes obtained after calcination at 750 °C (see Figure 2 for SEM). a) The spherical and amorphous Stoeber particles are ca. 150 ± 10 nm in diameter). b): HRTEM and SAED pattern of the crystalline oxidic ceria/zirconia material deposited in the interstices between the spherical amorphous silica particles during the spray-coating process. Inset in b): SAED pattern of the poly-crystalline, oxidic ceria/zirconia material deposited in the interstices between the SiO_2_ particles.

An examination of the tube surface composition by HRTEM reveals the spherical Stoeber sol particles of colloidal silica characteristic of an amorphous structure. They are closely linked by a film of nanocrystalline zirconia and ceria, which connects the individual spherical SiO_2_ particles (Figure 3). This is a result of the spray-coating procedure of the silica tubes with the ethanolic sol solution containing zirconia and ceria sol precursors. The spray-coating ‘stitches’ the sol–gel particles together and provides a very tight contact between them, which is essential for the mechanical stability of the ternary composite fibers compared to the bare SiO_2_ tubes (Figure 3b).

**Figure 4:**
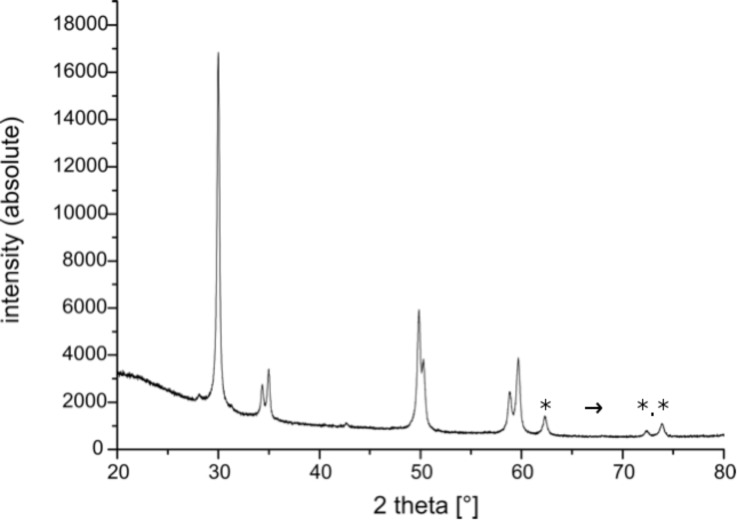
PXRD spectrum of oxidic microtubes of the composition Ce_0.13_Zr_0.87_O_2_@SiO_2_. Main diffraction peaks 2Φ° [(rel).: 29.968 [101] 999, 34.328 [002], 166, 34.977 [110], 200, 49.825 [112], 355, 321, 50.305 [200], 225, 58.819 [103], 225, 59.676 [211], 237. JCPDS # 88-2397 for Ce_0.12_/Zr_0.88_O_2_: 29.957 [101], 999; 34.320 [002], 83; 34.930, [110], 136; 49.778 [112], 321; 50.231 [200], 170, 58.787 [103], 113; 59.516 [211], 214. * not assigned.

Powder X-ray diffraction (PXRD, Figure 4) of the CeO_2_/ZrO_2_/Ce@SiO_2_ composite tubes corroborates the presence of one single crystalline phase which can be attributed to the tetragonal ZrO_2_ host lattice structure (JCPDS # 88-2397 for Ce_0.12_/Zr_0.88_O_2_, Ce/Zr ratio 0.13). This composition is very close to the Ce/Zr molar ratio of 0.15 employed in the sol–gel process and results in a calculated Ce_0.13_/Zr_0.87_O_2_ phase composition_._ No signals of crystalline phases of SiO_2_ were detected. The composition is in accordance with the molar ratio Ce/Zr employed in the synthetic spray-coating procedure. It is known that the substitution of Ce^4+^ (ionic radii = 111 pm) for Zr^4+^ (ionic radii = 98 pm) stabilizes the tetragonal ZrO_2_ structure [10]. As shown for the solid solution composition Ce_0.12_/Zr_0.88_O_2_ [10], the formation of the tetragonal over the stable thermodynamic high temperature mono-clinic phase of ZrO_2_ depends on the total energy *E*_t_ = *E*_l_ + γ*S*_m_, where *E*_I_ is the lattice energy, γ is the surface energy, and *S*_m_ is the molar surface. The two values of *E*_I_ are nearly identical for the monoclinic and tetragonal phase (11016 vs 11011 kJmol^−1^) [16], so the favorite formation of one over the other is mainly determined by their individual surface energies γ (11.3 × 10^−5^ Jcm^2^ vs 7.5–7.7 × 10^−5^ Jcm^2^) [17–18]. Consequently, the tetragonal phase is the more stable one for the solid solution Ce_0.13_/Zr_0.87_O_2_ due to its lower surface energy. Moreover, the incorporation of Ce^4+^ into its crystal lattice increases that effect.

**Figure 5:**
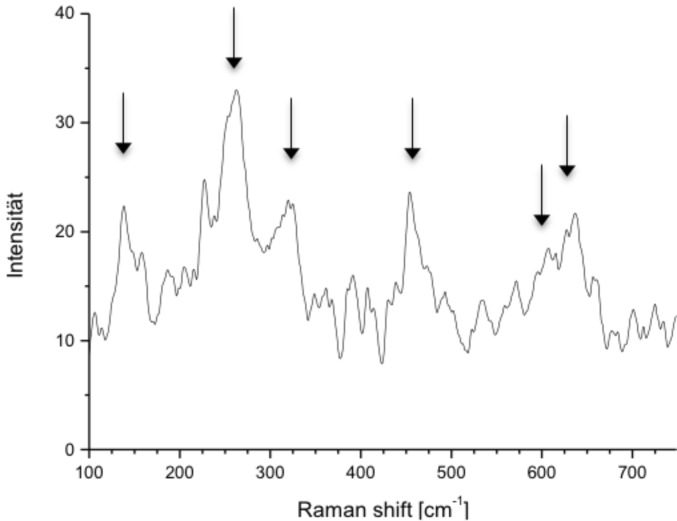
Raman spectrum of oxidic microtubes of composition Ce_0.13_Zr_0.87_O_2_@SiO_2_ displaying resonance signals at 146, 263, 313, 459, und 630 cm^−1^ as well as a shoulder at around 560 cm^−1^. The arrows indicate the position of these six signals [9].

In addition to the PXRD pattern, Raman spectroscopy allows for the identification of the phase composition of the Ce/ZrO_2_ solid solution of the composite microtubes (Figure 5). The tetragonal phase of ZrO_2_ in space group P4_2_/nmc is characterized by six active Raman modes [9,19–20], all of which are found for the Ce_0.13_/Zr_0.87_O_2_@SiO_2_ composite material (Figure 5, marked signals: A_1g_ + 2×B_1g_ + 3×E_g_). In contrast, lattice doped ZrO_2_ in the monoclinic phase gives just a single Raman resonance at 490 cm^−1^ [20–21]. Thus, a combination of Raman spectroscopy and PXRD facilitates the recognition of the lattice host structure of the composite tubes Ce_0.13_/Zr_0.87_O_2_@SiO_2_ as the tetragonal cubic structure of ZrO_2_.

**Figure 6:**
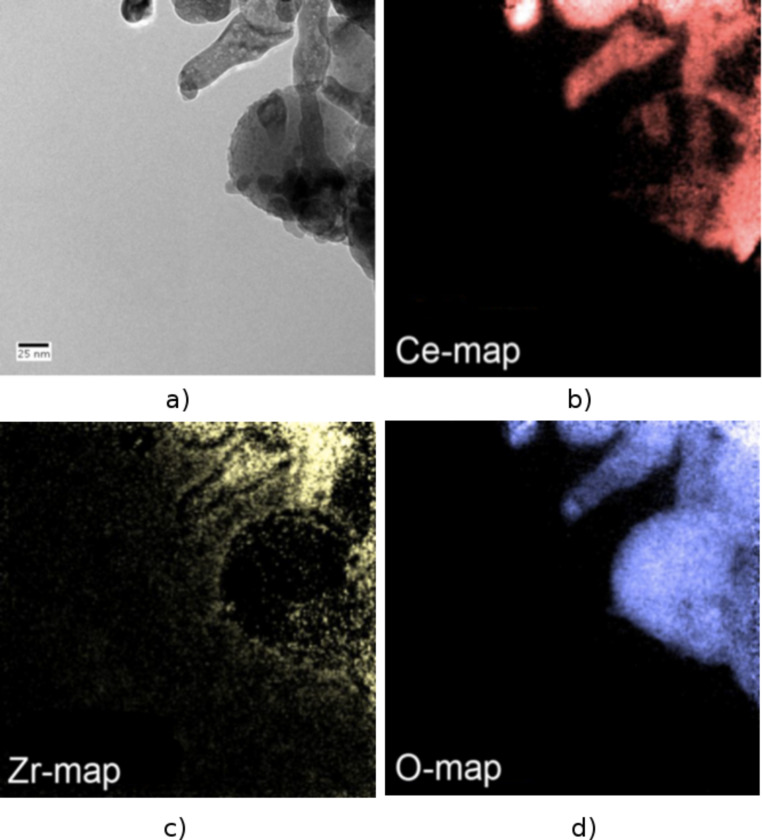
EFTEM, element mapping of calcined ternary oxide CeO_2_/ZrO_2_@SiO_2_ microtubes. a) TEM overview of the sample area scanned in the EFTEM experiment. b) Element mapping of cerium (red, Ce). c) Element mapping for zirconia (yellow, Zr). d) Oxygen map which is evenly distributed throughout the scanned sample area (blue, O).

EFTEM investigations on the CeO_2_/ZrO_2_@SiO_2_ microtubes material were performed in order to investigate the homogeneity of the Zr/Ce impregnation after final calcination as well as to gather information about the overall distribution of the oxides CeO_2_ and ZrO_2_ on the surface of the silica tubes. An element mapping analysis of the resulting mixed oxide tubes (Ce, Zr, and O) shows that oxygen is evenly distributed throughout the whole sample as would be expected from an all oxidic material. The majority of the cerium and zirconium are found on the surface of the spherical Stoeber particles, which indicates that, primarily, the intersticial volume between the silica particles is filled (see Figure 3 TEM and Figure 6 EFTEM). This fact becomes especially obvious when comparing the EFTEM Zr- and O-maps (Figure 6c,d).

**Figure 7:**
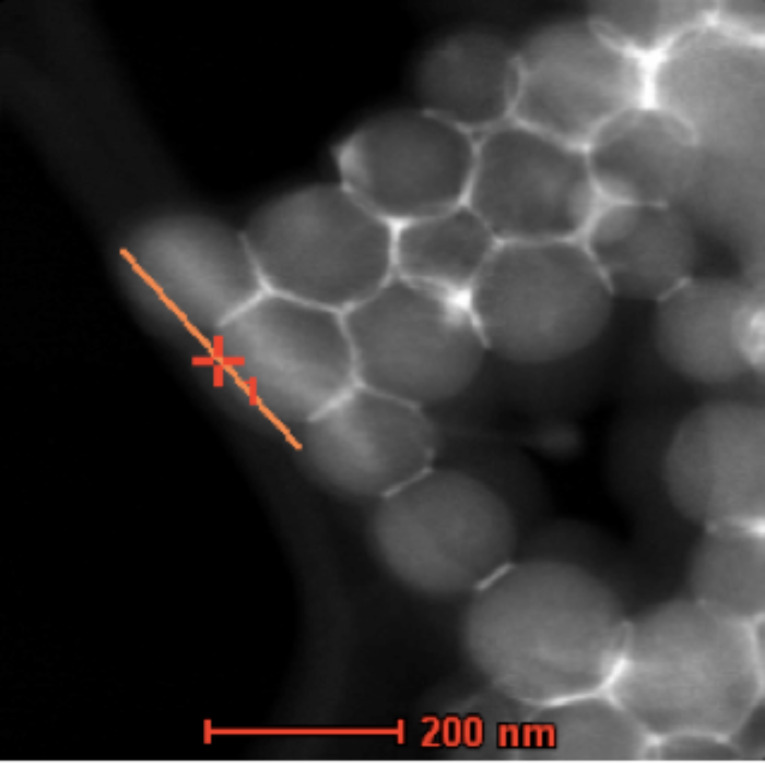
HAADF-STEM image of a line scan of a length of 200 nm . The red artificial marking line indicates the scan direction from left to lower right. The original line scan of the e-beam was in the middle of the Stoeber spheres (note the brighter contrast line running along the surface of the first three Stoeber spheres).

**Figure 8:**
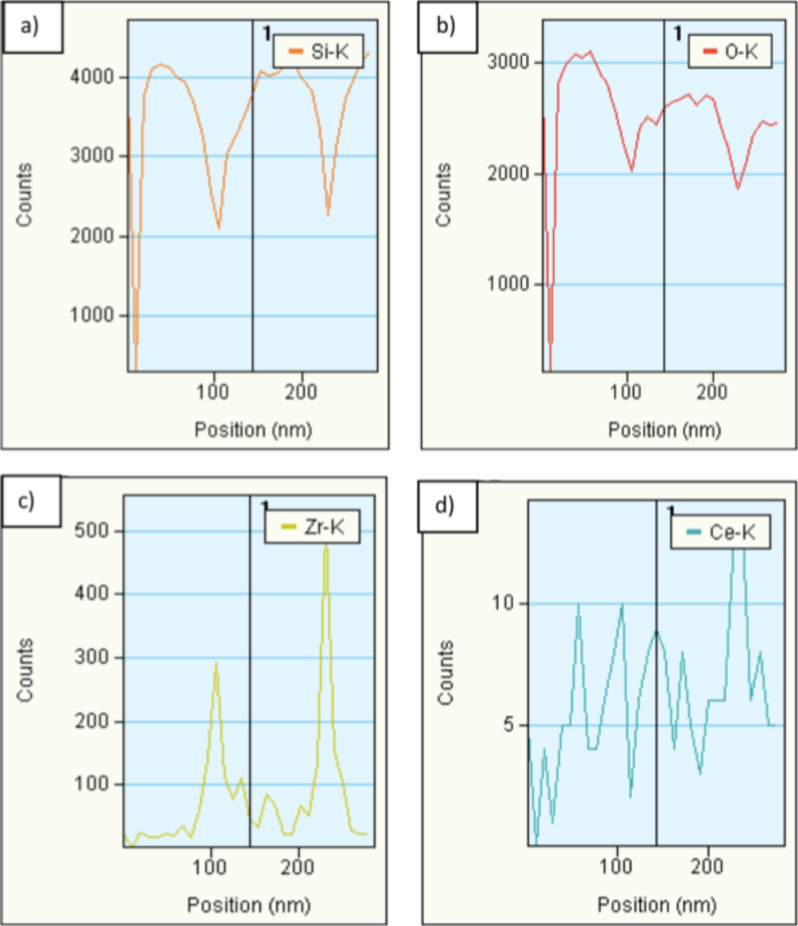
a–d) EFTEM, individual EDX profiles for the elements Si, O, Zr and Ce.

In order gain further analytical information on the ceria and zirconia distribution on the silica tubes we studied the elemental distribution of both elements on the nanometer scale by using EDX in STEM mode. Figure 7 shows the area an EDX line scan of the CeO_2_/ZrO_2_@SiO_2_ microtubes was performed (Figure 8) obtained from a HAADF STEM (high angle annular dark field imaging scanning electron microscopy) investigation. The STEM analysis proves the closely packed silica Stoeber spheres, which are interconnected by the solid solution of the composition Ce_0.13_/Zr_0.87_O_2_. The bright contrast visible in the dimples between the spheres indicates selective ceria/zirconia deposition. The EDX line scan (e-beam scan width <1 nm) allows the determination of the element specific distribution on the surface of the Stoeber spheres. The diameter of the silica spheres (150 ± 10 nm, see Figure 3) constituting the microtubes is reflected in the ratio of counts vs position in the Si-Kα and O-Kα spectra, which show for both elements a steep intensity increase between 110 and ca. 250 nm very well in line with the diameter of the silica spheres. According to the preferential deposition of the ceria/zirconia solid solution of composition Ce_0.13_/Zr_0.87_O_2_ in the dimples between individual silica spheres the corresponding intensity maxima of these elements are found between the spheres. In contrast, the zirconium and oxygen peak intensities have their respective minima between the spheres. This corroborates in an analytical way what has been assumed by HSEM and in HRTEM. Moreover, the intensity of cerium compared to zirconium in the elemental EDX scan is significantly lower, which reflects the phase composition as found by PXRD and Raman spectroscopy.

## Conclusion

A template-directed synthesis was employed for the synthesis of micrometer sized CeO_2_/ZrO_2_@SiO_2_ composite tubes. PS template fibers were obtained by electro-spinning and used to anchor dense silica spheres on their surface by a sol process, which consists of applying a sol solution infiltration process after plasma functionalization. The tethering of ceria and zirconia sols on the surface of PS/silica templates was achieved by spraying a sol solution containing ceria and zirconia sol precursors. Calcination removes the PS template and converts the dense fibers into a tube morphology. EFTEM investigations have proven the elemental distribution of cerium and zirconium on the surface of the ternary composite tubes, but interestingly they predominantly appeared in the interstices between separate silica spheres of the tubes. For future studies it may be worthwhile to investigate to what extent the allocation of the ceria–zirconia nanocrystallites situated mainly in the dimples (or grain boundaries) between the silica particles would increase the macroscopic fracture toughness of the ceramic composite. Such an effect has been shown for the grain boundaries of a composite of high purity silicon nitride and amorphous Y_2_O_3_ (2 wt %) [22]. Finally, it can be foreseen when applying higher ceria/zirconia sol concentrations in the spray coating process, even thicker oxide films with varying ration Ce*_x_*/Zr*_y_* could be obtained. These solid solutions could then cover the surface of silica tubes, resulting in an increase in the thickness of the overall tube walls. Therefore, our process allows to fine-tune the morphology of the ternary ceramic composition over a wider range. In summary, it can be concluded that the exo-templating process by means of electrospun polymeric fibers and inorganic oxides combined with the process of spray coating of sol particles onto structured one-dimensional objects presents a new synthetic route which is expected allow access to a broad variety of hierarchically structured multinary composites.

## Experimental

SEM measurements were performed on a FEI, XL30, FEG instrument. TEM and STEM investigations were carried out on a FEI Tecnai F20 G2 operated at 200 kV and equipped with a Gatan EDX detector system. Samples were dispersed in ethanol by ultrasonification for 10 min before a few drops of the dispersion were placed on lacey carbon grids. PXRD were measured on a StoeCIE, StadiP with Co Kα-1 radiation. Raman spectra were measured at a Senterra Raman microscope at 785 nm exitation wavelength. Electrospinning was carried out on a home-build electrospray setup under the specified conditions.

### Synthesis of silica microtubes and ceria/zirconia@silica composite microtubes

16 g polystyrene (PS) granules were added into 85 mL of a DMF/THF solution (2:1 in weight), and the mixture was stirred until complete dissolution of the PS. The obtained solution was electrosprayed through a nozzle with a longitude of 40 mm and a diameter of 0.8 mm at 26.8 kV and 25 cm electrode distance. Electrospinning under these conditions for several hours yielded dense PS faser mat which were used for the following experiments. Gas phase rf-plasma functionalization was performed in air at ambient temperature for 10 s.

60 mg of the electrospun plasma functionalized PS fibers (26.8 kV, 16 wt % PS, acetone/DMF = 60/40, Evonik-Röhm GmbH) were dispersed in 30 mL of ethanol. 1mL distilled water, 1mL tetra-ethoxysilane (TEOS, TEOS, ABCR, 98%) and 2.5 mL of 25% ammonia solution were added one at a time. The obtained mixture was vigorously stirred for 6 h. The PS/silica composite fiber mats were washed with ethanol and dried at 80 °C for 12 h in an oven. Final calcination was at 750 °C for 4 h.

### Ceria/zirconia@silica composite microtubes of composition Ce_0.13_/Zr_0.87_O_2_@SiO_2_

The PS/silica composite fibers were dried overnight at 80 °C and then used for the synthesis of the ternary composite microtubes. 0.91 g cerium(III)chloride (2.5 mmol, Alfa Aesar, 99%) and 5.26 g zirconium oxychloride (16.7 mmol, Alfa Aesar, 99%) were dissolved separately in 20 mL of ethanol. The obtained solutions were mixed, stirred at 75 °C for 30 min, and left standing for 48 h at room temperature for aging. This sol was then deposited by spray-coating on the fibrous PS/silica composite material. After drying for 12 h at 60 °C the composite material was finally calcined at 750 °C for 4 h (oven Heraeus Instr., thermocon P^®^).
